# Does COVID-19 Infection within 1 Week after Total Knee Arthroplasty Affect Patients’ Early Clinical Outcomes? A Matched Case–Control Study

**DOI:** 10.3390/jcm12134496

**Published:** 2023-07-05

**Authors:** Jung-Kwon Bae, Jae-Sung Seo, Seong-Kee Shin, Seo-Jin Kim, Jun-Ho Kim

**Affiliations:** 1 Department of Orthopedic Surgery, Seoul Medical Center, Seoul 02053, Republic of Korea; 2 Department of Orthopedic Surgery, Kyung Hee University Hospital at Gangdong, Seoul 05278, Republic of Korea

**Keywords:** COVID-19, knee, arthroplasty, outcomes, complications

## Abstract

Recent studies have reported the impact of previous COVID-19 infection on the early clinical outcome after total knee arthroplasty (TKA). However, the timing of infection before the surgery was not constant and a study on patients with COVID-19 infection within 1 week after TKA is lacking. This study aimed to determine the effect of COVID-19 infection within one week after TKA on the postoperative outcomes and to compare the early clinical outcomes to those who were not infected with COVID-19 before and after surgery. No significant differences were observed between the two groups in terms of clinical outcomes or complications. The length of the hospital stay (LOS) was significantly longer for the COVID-19 group than for the non-COVID-19 group (*p* < 0.05). The erythrocyte sedimentation rate (ESR) and C-reactive protein (CRP) levels were higher for the study group on postoperative days 9 and 12 than for those in the control group (*p* < 0.05). However, D-dimer levels were not significantly different between the two groups. We should cautiously consider that COVID-19 infection within 1 week after TKA may be associated with increased ESR, CRP levels, and length of hospital stay, but they are not associated with the worsening of early clinical outcomes or the occurrence of complications.

## 1. Introduction

The SARS-CoV-2 (COVID-19) virus has led to an unprecedented cancellation of elective surgeries and it is continuously challenging the previously established standards of healthcare institutions [1,2]. Total knee arthroplasty (TKA) is a widely performed elective surgery, with a global count of over one million procedures [3]. However, the COVID-19 pandemic had a significant impact on surgical volumes, leading to a substantial decline in TKA surgeries. After the first peak of the pandemic, healthcare organizations recognized the need for a planned return to routine healthcare [4,5]. With the implementation of perioperative protocols and the widespread administration of COVID-19 vaccinations, elective procedures have been safely reintroduced [6]. Restoring surgical volumes to pre-pandemic levels is particularly beneficial for individuals with knee osteoarthritis who have faced delays in receiving the necessary care. Prolonged delays in treatment have been associated with increased mortality rates and the development of additional healthcare complications [7]. Therefore, it is important to determine the impact of COVID-19 infection on the outcomes of TKA surgery. An expensive multinational study involving multiple research centers has revealed that COVID-19 is widely believed to increase the risk of systemic coagulation activation, leading to thrombotic disease and acute respiratory syndrome [5,6,7]. However, the effect of COVID-19 on the outcome of TKA remains debatable. Some studies have reported a major increase in venous thromboembolism (VTE) events in patients with TKA and recommend that all patients undergoing TKA should be assessed for the risk of VTE [8,9]. However, another study reported that a history of COVID-19 infection does not appear to increase the risk of VTE following primary TKA [10]. Furthermore, a recent study reported that COVID-19 did not adversely affect early complications or patient-reported outcomes measures (PROMs) after TKA [11]. However, all these studies were conducted on patients who had COVID-19 infection before TKA; further, the timing of infection before the surgery was not constant [8,9,10,11]. Moreover, the influence of COVID-19 infection on the body may decrease over time after infection [12]. Considering that the clinical presentation of COVID-19 starts within the first week [13,14], this study aimed to determine the effect of COVID-19 infection within 1 week after TKA on early outcomes and to compare these early clinical outcomes to those who were not infected with COVID-19 before or after surgery, by considering, specifically, PROMs, perioperative laboratory results, and early complications. Previous studies have not clearly determined when patients were infected with COVID-19, so it was difficult to accurately analyze the effect of COVID-19 infection on TKA. However, this study is able to provide more accurate analysis because the study was conducted by limiting the time of COVID-19 infection to within 1 week after TKA. We hypothesized that COVID-19 infection within 1 week after TKA would adversely affect PROMs, laboratory results, and early complications, such as thrombotic disease and acute respiratory symptom. In this retrospective study, PROMs as a primary outcome were assessed for the impact of COVID-19 infection. Secondary outcomes were laboratory results and complications. In the end, it aims to objectively identify how COVID-19 infection affects TKA and provide better information for orthopedic surgeons involved in TKA to cope with COVID-19 infection during patient treatment.

## 2. Materials and Methods

### 2.1. Study Design and Patients

**Figure 1:**
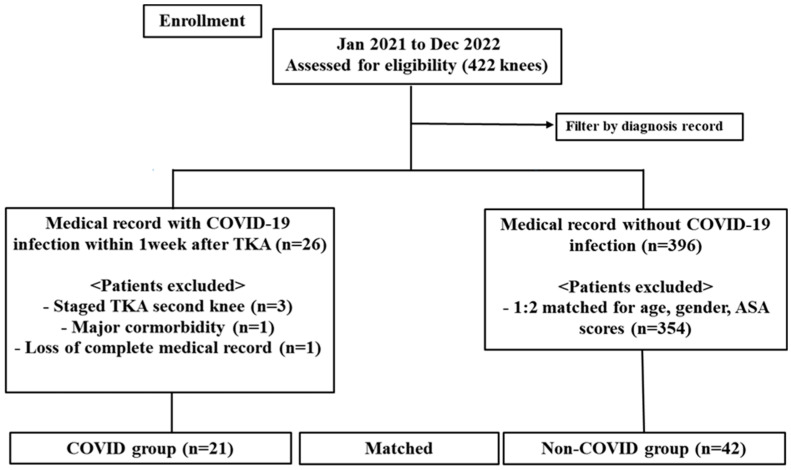
Flow diagram showing the number of knees that met the study criteria. Legend: TKA: total knee arthroplasty; ASA: American Society of Anesthesiologists; n: number of cases.

The present study retrospectively collected clinical data from consecutive patients who underwent TKA at our institute between January 2021 and December 2022. All patients who had COVID-19 infection within 1 week after TKA were identified. Patients with a history of COVID-19 infection before TKA were not included. Those with a second knee in staged bilateral TKA and secondary osteoarthritis (OA) were also excluded to reduce bias. The exclusion criteria included the presence of major comorbidities, such as severe pulmonary disease, coagulopathy, and a history of VTE disease. The final study cohort included 21 patients with TKAs (the COVID-19 group). To reduce the selection bias and potential confounding effects, a matched comparison cohort was subsequently created as the non-COVID-19 group. In the database of 422 TKAs conducted, we identified a comparison cohort with a ratio of 1:2. The patients were matched for sex, age, unilateral surgery, prosthesis type, same surgeon, same operating unit and surgical timing, and American Society of Anesthesiologists (ASA) score; we obtained 42 patients who underwent TKA and did not have a history of COVID-19 infection (the non-COVID-19 group) (Figure 1). The study protocol was approved by the institutional review board.

### 2.2. Surgical Methods and Postoperative Treatment

All the operations were performed by a single senior surgeon at a single institution. All the patients were managed using the same protocol. A pneumatic tourniquet was applied during the operation and deflated before wound closure. Cemented components were used in all the cases. The prostheses used were the posterior-stabilized Anthem System (Smith & Nephew, Memphis, TN, USA) in 41 knees and the Lospa system (Corentec, Cheonan, Korea) in 22 knees. There were 15 out of 41 cases with the Anthem System, and 6 out of 22 cases with the Lospa System of COVID-19 infection 1 week after TKA. The patella was not resurfaced in any of the cases [15,16]. A suction drain was placed on the knee joint and opened without clamping. Compressive elastic stockings were applied to all patients to prevent deep vein thrombosis for four weeks postoperatively. Routine antithrombotic prophylaxis was not used in consideration of drug-related bleeding complications [17,18]. Range of motion (ROM), calf pump exercises, straight leg raising, and bedside continuous passive mobilization were initiated on postoperative day 1. The suction drain was removed 24 h postoperatively and the patients were allowed to walk as tolerated.

### 2.3. Confirmed Criteria and Treatment of COVID-19 Infection

Regardless of clinical signs, cases in which infection was confirmed according to diagnostic criteria were defined as COVID-19 infection (diagnostic tests: COVID-19 real-time RT-PCR, virus isolation). In the COVID-19 patients presenting with fever (37.5 °C or higher) and/or respiratory symptoms (cough, sore throat, etc.) within 14 days of visiting China (including the special administrative regions of Hong Kong and Macau) or other COVID-19-affected countries (as listed on the WHO website), we implemented a treatment protocol of administering intravenous Remdesivir for a duration of 3–5 days [19]. Additionally, we adopted a standard approach for managing other symptoms similar to respiratory conditions. Subsequent to treatment, continuous X-ray monitoring was employed for tracking and observation, while routine CT scans were not conducted.

### 2.4. Clinical Evaluation and Complications

The American Knee Society (AKS) score and Western Ontario and MacMaster (WOMAC) were used for clinical evaluation [20,21]. Regular follow-up evaluations were performed at the outpatient department at 6 weeks, 3 months, 6 months, and 1 year after surgery. Postoperative clinical outcomes were collected at the latest follow-up. Any postoperative complications or readmissions within 90 days after discharge were recorded and compared between the groups. Any hemarthrosis, wound dehiscence, periprosthetic joint infection (PJI), deep vein thrombosis (DVT), or stiffness were recorded. The patellar circumference was evaluated for hemarthrosis. Acute postoperative PJI was diagnosed based on the ICM from 2018 [22]. The incidence of venous thromboembolism (DVT and symptomatic pulmonary embolism) was evaluated using computed tomography (CT) venography before discharge. For the COVID-19 group, any shortness of breath, chest pain, or blood-streaked sputum suggestive of pulmonary embolism were evaluated using a pulmonary thromboembolism CT [23]. Stiffness was defined by using a cut-off of approximately 95° of flexion that prevented the patient from performing most of the activity [24].

### 2.5. Laboratory Evaluation

Blood samples were obtained before surgery and on the first, second, fifth, ninth, and twelfth postoperative days (POD), from the patients who underwent unilateral TKA. D-dimer levels were measured from the samples collected between preoperative screening day 7 and 14 and on the twelfth postoperative day. The hematologic parameters compared between the two groups were hemoglobin (Hb) levels, erythrocyte sedimentation rate (ESR), C-reactive protein (CRP) levels, and D-dimer levels. CRP levels were measured using the turbidimetric immunoassay on the COBAS c 702 (Roche, Basel, Switzerland) and the normal range was set to less than 0.4 mg/dL. ESR was measured using a photometric kinetic analysis of capillary-stopped flow on the Test-1 system (Alifax, Padova, Italy); the normal range for the laboratory at our hospital was less than 20 mm/h. D-dimer levels were measured using a Stago STA-R MAX (Diagnostica Stago, Mt Olive, NJ, USA) that used a turbidimetric immunoassay and the normal range was less than 490 ng/mL.

### 2.6. Statistical Analysis

Sample size calculations were performed using R (version 3.5.1, R Foundation for Statistical Computing, Vienna, Austria). The sample size was evaluated based on the measured primary outcome and was calculated to be 13 knees in the study group and 26 knees in the control group, assuming 80% power, an alpha error of 0.05, and an enrollment ratio of 2. A difference of 26% in the clinical scores was selected based on calculations of effect sizes from values of the postoperative clinical score reported in a retrospective study of clinical outcomes in patients with TKA [8]. The Kolmogorov—Smirnov test was used to evaluate whether the variables followed a normal distribution. The independent samples *t*-test was used to compare the continuous data. A chi-square or Fisher’s exact test was used to compare categorical data. The statistical significance was set at a two-sided *p*-value of less than 0.05. All analyses were performed using SPSS software version 23 (IBM Corp, Armonk, NY, USA) and continuous data are expressed as the mean± standard deviation. 

## 3. Results

### 3.1. Clinical Evaluation

No significant demographic differences were observed between the COVID-19 and non-COVID-19 groups, except for the length of hospital stay (LOS) (Table 1). The mean AKS and WOMAC scores were not significantly different between the two groups. The average ROM also did not differ between the groups (Table 2).

### 3.2. Perioperative Laboratory Results

**Figure 2:**
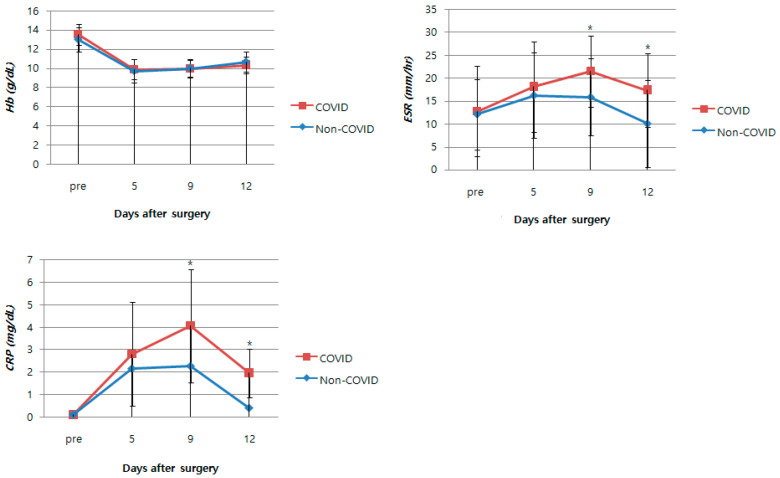
Serial changes of Hb, ESR, and CRP. Data are presented as the mean ± standard deviation. Values with statistical significance are marked with * for the comparisons between COVID-19 and non-COVID-19 groups. Legend: Hb: hemoglobin; ESR: erythrocyte sedimentation rate; CRP: C-reactive protein.

The mean preoperative Hb, ESR, and CRP levels did not differ significantly between the groups. The levels for all three serum tests, preoperatively and on POD 5, 9, and 12, are presented in Table 3 (Figure 2). The Hb levels were not significantly different between the two groups during hospitalization. The ESR and CRP levels showed no differences between the groups on POD 5. However, a significant difference was observed between POD 9 and 12. The D-dimer levels did not differ between the two groups on POD 12 (Table 4).

### 3.3. Complications

No significant differences were observed in complications, such as hemarthrosis, stiffness, wound dehiscence, PJI, pneumonia, DVT, and PE, between the two groups (Table 5). The 90-days readmission occurred for one knee in both groups: cellulitis (*n* = 1) in the COVID-19 group and gastroenteritis (*n* = 1) in the non-COVID-19 group.

## 4. Discussion

The main finding of the present study is that COVID-19 infection within 1 week after TKA does not affect early clinical outcomes and complications. However, it may be associated with increased ESR and CRP levels and length of hospital stay, but they are not associated with worsening early clinical outcomes and the occurrence of complications. Considering the potential systemic effects of COVID-19 on patients, it is important to acknowledge the findings of our study, which indicate that the occurrence of infection, pneumonia, and other complications are similar between patients with and without a history of COVID-19 infection. Although further research is necessary to establish a causal relationship, these findings deserve careful consideration by physicians who are providing care to TKA patients with a history of COVID-19 infection. Furthermore, it is important to recognize that the various COVID-19 variants and sub-variants could potentially influence the disease’s impact on patients and subsequently affect outcomes such as pneumonia and DVTs.

COVID-19 created an unprecedented challenge for elective orthopedic care [1,2]. Several studies have reported that patients with COVID-19 undergoing surgery have a higher rate of mortality and lung complications [25,26,27,28]. Patrick et al. [8] reported that the COVID-19 pandemic negatively affected the early clinical outcome parameters of elective primary TKA. However, this study was conducted only in the presence or absence of COVID-19 before surgery, with no mention of the timing of the infection. Moreover, previous studies have reported that the mean time from symptom onset to hospitalization varied from 2 to 5 days [13,14]. Because the onset of overt clinical symptoms, such as fever, dyspnea, and signs of pneumonia, usually occurs 5–7 days after the initial symptom onset [14], the present study was conducted by clearly limiting the timing of COVID-19 infection. Moreover, no study has demonstrated the effect of COVID-19 infection within 1 week after TKA on early clinical outcomes and complications. Recent studies have reported that previous COVID-19 infections do not adversely PROMs or complications of primary TKA [10,11].The findings of the current study are consistent with those of previous studies and indicate no significant difference in PROMs between the two groups, even if infection occurred within 1 week after TKA. Furthermore, although previous studies have shown a higher incidence of postoperative complications after TKA for previous COVID-19 infection [29,30], in our study, no difference was found between the groups in terms of pneumonia and other significant complications. We believe that these results are due to the close treatment of the COVID-19 group with longer hospitalizations.

ESR and CRP are acute-phase reactants that reflect a measure of the acute-phase response after a stimulus and have been widely used to monitor the postoperative course after TKA because of their advantageous characteristics, including a rapid increase in concentration, relatively short lag time, and cost-effectiveness [31,32,33]. Park et al. [34] reported that CRP levels increase rapidly, reaching a peak on the second day. ESR levels peak on the fifth day and then gradually decrease. Ibrahim et al. [35] reported that the highest mean ESR and CRP values occurred on days three and five, respectively. Similarly, the current study shows that ESR and CRP levels peaked on day five in the non-COVID-19 group. However, the proper interpretation of ESR and CRP levels is not easy, especially when COVID-19 infection occurs during the postoperative recovery period. To obtain helpful guidelines for interpretation, this study sought to determine whether ESR and CRP levels differed between the COVID-19 and non-COVID-19 groups when COVID-19 infection occurred within 1 week after TKA. Our results show that ESR and CRP levels tended to increase without decreasing within 1 week in the COVID-19 group. Although the acute-phase response from TKA decreased over time, ESR and CRP levels did not change to a decreasing trend after 1 week, as the COVID-19 infection affected the new acute reaction.

Sun et al. [36] reported that hemoglobin levels in patients infected with COVID-19 are lower than those of the general population. However, there was no difference in the preoperative Hb between the COVID-19 group and the non-COVID-19 group in the present study because this study was conducted on patients infected with COVID-19 within one week after surgery. Anna et al. [37] reported that COVID-19 infection did not have an impact on the overall drop in hemoglobin levels or transfusion requirements after elective TKA. In addition, although ferritin levels were significantly different between the two groups, there was no evidence that the COVID-19 group’s hemoglobin levels recovered more slowly within the first four–six weeks after surgery. The findings of our study are consistent with those of previous studies and indicate no significant difference in postoperative Hb between the COVID-19 group and the non-COVID-19 group. Even if there was a COVID-19 infection within 1 week after surgery, there was no significant effect on the decreasing tendency and recovery ability of Hb.

D-dimer is a widely available serum biomarker known for its diagnostic utility for fibrinolytic activities in thromboembolic events [38,39]. Since its introduction in the 1990s, D-dimer concentration measurement has become an important test in patients with suspected thrombotic disorders. In general, the plasma D-dimer level is a sensitive but nonspecific marker of DVT, making it a good “rule-out” test with appropriate pretest probability [40,41]; elevated D-dimer levels represent a risk factor for DVT in patients with TKA [42]. The natural course of D-dimer levels after primary TKA has been reported in a few studies [43,44]. The results of our current study are similar to those of previous studies. On postoperative day 12, D-dimer levels remained 3.5 times as high as their preoperative value for most patients. Meanwhile, D-dimer levels might be elevated in patients with active COVID-19 who are at risk for thromboembolic events [45,46,47]. It was unknown how D-dimer levels in the group infected with COVID-19 within 1 week after TKA differ from those of general patients undergoing TKA. Therefore, our study regularly obtained D-dimer levels on postoperative day 12 and confirmed that there was no difference between the two groups. Although the sample size was small, there was no difference in thromboembolic events between the two groups in terms of 90-day medical complications. These results suggest that an additional thromboembolic effect does not occur even if COVID-19 infection occurs after TKA.

This present study found that patients who had a COVID-19 infection within 1 week after TKA did not exhibit significantly higher rates of postoperative complications compared to the non-COVID-19 group. Specifically, there were no significant differences in the occurrence of hemarthrosis, stiffness, wound dehiscence, pneumonia, PJI, and 90-day readmission between the two groups. These findings are important as they suggest that the timing of COVID-19 infection in relation to TKA does not significantly impact the occurrence of common complications associated with the surgery. Patients undergoing TKA may vary in age and have different comorbidities that can influence the likelihood of unplanned hospital readmissions following surgery. Therefore, it is crucial for healthcare providers to identify patients who are at higher risk for such complications in order to minimize their occurrence and evaluate outcomes based on risk stratification. According to a study by William et al. [48], the readmission rate within 90 days after primary TKA was reported to be 6%. In our study, the 90-day unplanned readmission rate in the non-COVID-19 group was found to be 2.4%, which aligns with similar findings in the existing literature. Even in the COVID-19 group, the readmission rate of 4.6% was consistent with previous reports. This information is important for physicians and healthcare providers involved in the care of TKA patients with a history of COVID-19 infection, as it reassures them that the risk of postoperative morbidity and complications is comparable to patients without a prior COVID-19 infection.

Despite these informative results, our study has some limitations. First, it was a retrospectively study and a major challenge was obtaining the largest dataset possible to fully validate our hypothesis. However, the number of patients included in this study surpassed the requirement outlined in the power analysis. Considering the rare incidence of COVID-19 infection within one week after TKA, it would be difficult to conduct a prospective study with a more sufficient number of patients. Second, two different prostheses were used. However, our study was conducted by a single surgeon at a single institute and we used posterior-stabilized knee designs in all the patients without patella resurfacing to reduce bias. Finally, we acknowledge that the discrepancy in the length of hospital stays for the non-COVID-19 group can be attributed to the necessity of extended hospitalization for patients with COVID-19. Nevertheless, our study has important strengths. There have been no reports of patients with COVID-19 infection during the recovery period within 1 week after TKA. As mentioned, the timing of infection before surgery was not constant in previous studies. The results under the precise conditions of this study will help healthcare professionals to evaluate the progress of rehabilitation. They can determine if the patient is responding well to physical therapy and rehabilitation exercises, and make any necessary adjustments to the treatment plan.

## 5. Conclusions

We should cautiously consider that COVID-19 infection 1 week after TKA may be associated with increased ESR and CRP levels and length of hospital stay, but they are not associated with a worsening of early clinical outcomes or the occurrence of complications. This study reports that having a history of COVID-19 infection should not significantly increase our concern of their perioperative risk. Therefore, if treated according to the established protocol, we can achieve outcomes as favorable as those for patients who have not had COVID-19 infection.

## Figures and Tables

**Table 1:** Patients’ demographic characteristics.

Variables	COVID-19 Group (*n* = 21)	Non-COVID-19 Group (*n* = 42)	*p*-Value ^a^
Sex (male/female) ^b^	3/18	5/37	0.789
Mean age (years) ^c^	72.8 ± 7.9	74.9 ± 6.5	0.252
Mean BMI (kg/m^2^) ^c^	27.9 ± 5.8	26.6 ± 4.6	0.312
Mean ASA score ^c^	2.1 ± 0.4	2.0 ± 0.3	0.618
Preoperative HKA angle (°) ^c^	−8.3 ± 4.2	−7.7 ± 6.0	0.661
Preoperative K–L grade (3/4) ^b^	2/19	5/37	0.776
Length of hospital stay (days) ^c^	17.1 ± 1.4	13.0 ± 0.6	<0.001
Follow-up period (months) ^c^	10.6 ± 7.5	10.7 ± 8.4	0.968

*n*: number of cases; BMI: body mass index; ASA: American Society of Anesthesiologists; HKA: hip–knee–ankle angle (negative values indicate varus alignment); K–L grade: Kellgren–Lawrence grade. ^a^ Student’s *t*-test was used to compare continuous variable outcomes between groups. ^b^ Data are presented as numbers. ^c^ Data are presented as means ± standard deviations.

**Table 2:** Clinical outcomes between the groups.

	COVID-19 Group (*n* = 21)	Non-COVID-19 Group (*n* = 42)	*p*-Value ^a^
*Preoperative*			
AKS knee score ^b^	30.8 ± 10.6	31.4 ± 10.4	0.846
AKS function score ^b^	32.9 ± 14.9	30.4 ± 12.9	0.485
WOMAC score ^b^	79.5 ± 3.2	80.9 ± 4.1	0.156
FC (°) ^b^	9.3 ± 7.8	8.5 ± 7.0	0.670
FF (°) ^b^	123.1 ± 11.8	125.8 ± 6.8	0.246
*Postoperative*			
AKS knee score ^b^	93.9 ± 3.3	92.7 ± 4.6	0.325
AKS function score ^b^	89.3 ± 6.0	91.6 ± 5.1	0.117
WOMAC score ^b^	20.0 ± 4.2	20.9 ± 4.2	0.413
FC (°) ^b^	2.1 ± 3.0	1.7 ± 3.1	0.559
FF (°) ^b^	128.3 ± 4.0	129.3 ± 1.8	0.192

*n*: number of cases; AKS: American Knee Society knee score; WOMAC: Western Ontario and MacMaster score; FC: flexion contracture; FF: further flexion. ^a^ Student’s *t*-test was used to compare continuous variable outcomes between groups. ^b^ Data are presented as means ± standard deviations.

**Table 3:** Comparison of perioperative laboratory results between the groups.

	COVID-19 Group (*n* = 21)	Non-COVID-19 Group (*n* = 42)	*p*-Value ^a^
*Hb (g/dL)*			
Preoperative ^b^	13.5 ± 1.1	13.0 ± 1.3	0.126
PO fifth day ^b^	9.9 ± 1.1	9.7 ± 1.2	0.585
PO ninth day ^b^	9.9 ± 0.9	10.0 ± 0.9	0.875
PO twelfth day ^b^	10.4 ± 0.9	10.7 ± 1.1	0.242
*ESR (mm/hr)* ^b^			
Preoperative ^b^	12.8 ± 9.8	12.1 ± 7.7	0.769
PO fifth day ^b^	18.2 ± 9.9	16.3 ± 9.4	0.457
PO ninth day ^b^	21.5 ± 7.8	15.9 ± 8.5	0.013
PO twelfth day ^b^	17.4 ± 8.0	10.1 ± 9.5	0.003
*CRP (mg/dL)*			
Preoperative ^b^	0.1 ± 0.1	0.1 ± 0.1	0.832
PO fifth day ^b^	2.8 ± 2.3	2.2 ± 2.1	0.280
PO ninth day ^b^	4.1 ± 2.5	2.3 ± 1.9	0.003
PO twelfth day ^b^	2.0 ± 1.1	0.4 ± 0.4	<0.001

*n*: number of cases; PO: postoperative; ESR: erythrocyte sedimentation rate; CRP: C-reactive protein. ^a^ Student’s *t*-test was used to compare continuous variable outcomes between groups. ^b^ Data are presented as means ± standard deviations.

**Table 4:** Comparison of perioperative D-dimer between the groups.

	COVID-19 Group	Non-COVID-19 Group	*p*-Value ^a^
Preoperative (ng/mL) ^b^	496 ± 159	482 ± 206	0.795
Postoperative 12 days (ng/mL) ^b^	1906 ± 259	1866 ± 288	0.595

ng/mL: Nanograms (10^−9^ grams) per milliliter. ^a^ Student’s *t*-test was used to compare continuous variable outcomes between groups. ^b^ Data are presented as means ± standard deviations.

**Table 5:** Comparison of 90-day medical complications between the groups.

Variables	COVID-19 Group (*n* = 21 Knees)	Non-COVID-19 Group (*n* = 42 Knees)	*p*-Value ^a^
Hemarthrosis ^b^	0(0)	0(0)	1
Stiffness ^b^	0(0)	0(0)	1
Wound dehiscence ^b^	0(0)	1(2.4)	1
Pneumonia ^b^	0(0)	0(0)	1
PJI ^b^	0(0)	0(0)	1
Total DVT ^b^	2(9.5)	3(7.1)	0.741
Proximal DVT ^b^	0(0)	0(0)	1
Symptomatic PE ^b^	0(0)	0(0)	1
90-days readmission ^b^	1(4.8)	1(2.4)	0.611

*n*: number of cases; PJI: periprosthetic joint infection; DVT: deep vein thrombosis; PE: pulmonary embolism. ^a^ Chi-square test or Fisher’s exact test. ^b^ Values are presented as a number (%).

## Data Availability

The datasets generated and/or analyzed during the current study are not publicly available due to the participants not consenting to the public data release of their data; however, they are available from the corresponding author on reasonable request.
